# Divergent axial morphogenesis and early *shh* expression in vertebrate prospective floor plate

**DOI:** 10.1186/s13227-017-0090-x

**Published:** 2018-01-31

**Authors:** Stanislav Kremnyov, Kristine Henningfeld, Christoph Viebahn, Nikoloz Tsikolia

**Affiliations:** 1 Department of Embryology, Faculty of Biology, Lomonosov State University Moscow, Leninskie Gory, 1, Builung 12, Moscow, Russia 119234; 2 Koltzov Institute of Developmental Biology, Russian Academy of Sciences, Vavilova Str., 26, Moscow, Russia 119991; 3 Center for Nanoscale Microscopy and Molecular Physiology of the Brain (CNMPB), Institute of Developmental Biochemistry, University Medical Center Göttingen, Justus-von-Liebig-Weg 11, 37077 Göttingen, Germany; 4 Institute of Anatomy and Embryology, University Medical Center Göttingen, Kreuzbergring 36, 37085 Göttingen, Germany

**Keywords:** Evo–devo, Gastrulation, Induction, Neural tube, Notochord, Sonic hedgehog, Vertebrates

## Abstract

**Background:**

The notochord has organizer properties and is required for floor plate induction and dorsoventral patterning of the neural tube. This activity has been attributed to sonic hedgehog (shh) signaling, which originates in the notochord, forms a gradient, and autoinduces *shh* expression in the floor plate. However, reported data are inconsistent and the spatiotemporal development of the relevant *shh* expression domains has not been studied in detail. We therefore studied the expression dynamics of *shh* in rabbit, chicken and *Xenopus laevis* embryos (as well as *indian hedgehog* and *desert hedgehog* as possible alternative functional candidates in the chicken).

**Results:**

Our analysis reveals a markedly divergent pattern within these vertebrates: whereas in the rabbit *shh* is first expressed in the notochord and its floor plate domain is then induced during subsequent somitogenesis stages, in the chick embryo *shh* is expressed in the prospective neuroectoderm prior to the notochord formation and, interestingly, prior to mesoderm immigration. Neither indian hedgehog nor desert hedgehog are expressed in these midline structures although mRNA of both genes was detected in other structures of the early chick embryo. In *X*. *laevis*, *shh* is expressed at the beginning of gastrulation in a distinct area dorsal to the dorsal blastopore lip and adjacent to the prospective neuroectoderm, whereas the floor plate expresses *shh* at the end of gastrulation.

**Conclusions:**

While *shh* expression patterns in rabbit and *X*. *laevis* embryos are roughly compatible with the classical view of “ventral to dorsal induction” of the floor plate, the early *shh* expression in the chick floor plate challenges this model. Intriguingly, this alternative sequence of domain induction is related to the asymmetrical morphogenesis of the primitive node and other axial organs in the chick. Our results indicate that the floor plate in *X*. *laevis* and chick embryos may be initially induced by planar interaction within the ectoderm or epiblast. Furthermore, we propose that the mode of the floor plate induction adapts to the variant topography of interacting tissues during gastrulation and notochord formation and thereby reveals evolutionary plasticity of early embryonic induction.

**Electronic supplementary material:**

The online version of this article (10.1186/s13227-017-0090-x) contains supplementary material, which is available to authorized users.

## Background

The brain and spinal cord display characteristic functional and structural differences along the dorsoventral axis. Differences are derived from unique combinatorial gene expression in the progenitor domains of the neural tube, which leads to development of distinct types of neurons [26]. The unique gene expression in the progenitor domains is suggested to be the result of fine-tuned signaling activities related to gradients of secreted morphogens [13]. Sonic hedgehog (shh) forms a ventral-to-dorsal gradient and is expressed in the floor plate and the notochord. The shh gradient in turn induces a gradient of GliA and GliB transcriptional factor activity, which together with Sox2 participates in the formation of distinct gene expression domains [53]. Similarly, Wnt and Bmp signaling is active from the dorsal pole of the neural tube and from the superficial ectoderm [12, 40, 44].

The initial dorsoventral patterning of the neural tube exemplifies a classical induction from neighboring tissue. As inductive interactions belong to central concepts in developmental biology [76], the early role of the notochord serves as a paradigmatic example of induction by organizers, which are defined by their ability to induce and pattern adjacent tissue [3]. Early studies in amphibian embryos suggested that the notochord is required for correct morphological dorsoventral patterning of the neural tube [37], while other observations indicated, however, that a more cautious interpretation is needed [38, 75]. Similarly, the notochord in the chick was shown to be required for floor plate development as well for its specific inductive function [59, 90]. For example, implantation of the notochord lateral to the neural tube causes ventralization of the lateral tube wall and formation of ectopic floor plate, as seen by cell shape change or ectopic axon outgrowth [59, 91].

*Shh* has been shown to be strongly expressed both in the notochord and in the floor plate of different organisms and to have the ability to induce a floor plate when expressed ectopically [18, 31, 65]. Importantly, an isolated *shh*-expressing floor plate forms a gradient of shh within the neural tube and is also able to induce a ventral fate in the adjacent neural tissue [60], which indicates that the floor plate also has organizer activity [3]. Therefore, the role of the notochord may be related to expression and secretion of shh, which induces its own expression in prospective floor plate cells. According to this hypothesis, *shh* expression in the notochord must precede its expression in the floor plate and, indeed, this has been unambiguously shown in mouse [10, 18] and rat [65] embryos. It has been suggested that the six progenitor domains responsible for dorsoventral differences in the ventral neural tube are induced even prior to *shh* expression in the floor plate [64] and, therefore, if the shh gradient is causally involved in this induction, the notochord must be the primary source of the gradient.

However, the universality of the notochord’s organizer role is under debate [36, 50]. In zebrafish, for example, neither mechanical ablation of the notochord progenitors [71] nor disturbed notochord development in *no tail* (*ntl*) mutants [28] inhibits floor plate development. Moreover, it was shown that hedgehog signaling is not required for development of the medial floor plate, but is involved in development of so-called lateral floor plate [48, 67]. Similarly, in *Xenopus laevis,* shh signaling has a minor influence on medial floor plate markers, while a strong suppression of the lateral floor plate marker *nkx2.2* occurs upon inhibition of shh signaling [54]. Although chicken embryos remain a classical model organism for floor plate induction [14, 63], the situation here is still complex: Replacement of the chicken node at 5–6 somite stages by their quail counterpart shows that both the floor plate and the notochord caudal to the explant develop from the node, while in situ analysis has shown that both structures express continuously HNF3 beta [85]. Based on shared progeny and gene expression as well as on results from experiments with notochord excision in 10–25 somite stages [85], Nicole Le Douarine and colleagues suggested that the notochord is *not* required for the chick floor plate induction [36, 85]. However, this conclusion was criticized [58, 63]. It was argued that the shared progeny and HNF3 beta expression is not relevant since node cells which become floor plate immediately lose *shh* expression within the neural environment and the expression domain is then de novo induced by shh protein secreted from the notochord [58]. James Briscoe and colleagues also argue in favor of the model assuming floor plate specification by shh from the notochord in amniotes [63]: inhibition of hedgehog signaling in both mouse and chick embryos impairs the floor plate identity only if the downregulation of hedgehog signaling occurs prior to 5–10 somite stages. Hence, floor plate induction by the notochord may occur earlier as previously believed and the results provided by the group of Nicole Le Douarine concern stages with already induced floor plate and cannot challenge the role of the notochord in the floor plate induction [63]. Further investigations [50] led to a modified model which postulates that a prolonged signal from the notochord is required for the posterior floor plate regions, which includes hindbrain and spinal cord and starts to form at stage 5, whereas the induction of anterior floor plate regions is caused by migrating prechordal plate mesoderm. Partial ablation of the chick notochord shortly after the beginning of its formation (HH stage 5 −) does not suppress differentiation of anterior (mid- and forebrain) floor plate as seen by *shh* expression. This model suggests also involvement of nodal signaling in anterior floor plate induction [50].

Remarkably, in spite of the controversy about the source of the signal leading to floor plate induction, the early expression domains of *shh* in the chick embryo have not been studied in detail to date in the tissues and stages involved. As shown in a different context, *shh* is expressed prior to the notochord formation in the area of the primitive node already at stage 4 [17, 39]. At this stage, *shh* displays a dense symmetrical domain in both node ectoderm and mesoderm of the primitive pit, in the neuroectoderm just anterior to the node as well as a scattered expression in the emerging mesoderm anterior to the node [86]. Furthermore, our previous studies suggested a left–right asymmetry of the chick axial organ formation. Whereas the notochord forms from the right node shoulder, the floor plate exhibits tissue continuity with the left side of the primitive node [49] and analysis of histological sections revealed an unexpected localization of *shh* expression in the early floor plate. Therefore, we studied in detail the succession of *shh* expression in the node, prechordal mesoderm, notochord and floor plate of the chick embryo. As *shh* expression in the chick differs from spatiotemporal dynamics of *shh* expression in mammals as exemplified by the early mouse embryonic cylinder, the need arose to clarify a possible evolutionary divergence of floor plate induction and we therefore analyzed *shh* expression at comparable stages in rabbit embryos (displaying a flat embryonic disk—in contrast to the mouse—typical for most mammals [78, 92]), as well in (anamniote) *X*. *laevis* embryos. As the expression of hedgehog family members Indian hedgehog and desert hedgehog displays highly divergent patterns at early stages in zebrafish, Xenopus, mouse and rabbit [8, 19, 20, 97], we also studied their expression in the chick. The results of this study challenge the model of “the ventral to dorsal induction” of *shh* expression [89] in the midline and indicate an evolutionary divergence of floor plate induction, which may explain the contradictory data mentioned above. Interestingly, early floor plate induction in the chick embryo is in line with data showing that neural induction in the chick starts even prior to gastrulation by precursor cells of the organizer [81]. Our comparative analysis of spatiotemporal succession of *shh* expression at perigastrulation stages may have an impact on evolutionary scenarios of dorsoventral patterning and pave the way for further functional studies on the notochord’s organizer function. Last but not least, our analysis is important because the “notochord first” view of *shh* expression is taken for granted in most recent publications and is widely presented in textbooks of developmental biology.

## Materials and methods

Fertilized white leghorn chicken eggs were incubated under humidified conditions at 38 °C for 6–40 h until embryos reached stages between 2 and 9 (see [86]). To collect chick embryos, the eggshell was opened and the embryos were prefixed in fixative. After excision of blastoderm, embryos were transferred into a Petri dish with warm Locke’s solution, rinsed to remove adherent yolk particles and fixed in 4% PFA in phosphate-buffered saline (PBS) for 1 h. Uterine horns of New Zealand white rabbits were removed and transferred into warm (37 °C) phosphate-buffered saline (PBS). Blastocysts were excised from endometrium, and the blastodisk was isolated and fixed in 4% PFA for 1 h (see [69]). Fixed chicken and rabbit embryos were washed several times in PBS, dehydrated by ascending alcohol concentrations and stored in ethanol at − 20 °C. For in situ hybridization of chicken and rabbit embryos, selected embryos were transferred to nylon baskets, rehydrated, treated with 10 mg/ml proteinase K (Roche, Grenzach-Wyhlen, Germany) in PBT for 10 min and postfixed in 0.2% glutaraldehyde/PBT for 20 min. For the prehybridization and hybridization, the baskets with embryonic disk were transferred to sterile screw-top PVC tubes (Bibby Sterilin, Staffordshire, UK). After 1 h prehybridization at 70 °C in a heating block in hybridization buffer [50% formamide, 1.4X SSC, 0.1% 0.5 mM EDTA, 50 µg/ml t-RNA, 0.2% Tween-20, 0.5% CHAPS, 50 µg/ml heparin (AppliChem, Darmstadt, Germany)], the embryos were hybridized overnight at 70 °C in hybridization buffer with 1 µg/ml digoxygenin-labeled cRNA denatured at 95 °C. Labeled cRNA of chicken and rabbit shh and patched 2 was synthesized by in vitro transcription from PCR products of previously published plasmid DNA [2, 25, 39, 51] or from PCR product of a synthetically produced probe corresponding to bp 911–1714 of chicken dhh (GenBank accession number: XM_015300320.1) and 729–1405 bp of chicken ihh (GenBank accession number: NM_204957.2) was obtained from BioCat (Heidelberg, Germany). In the next step, embryos were washed in prewarmed hybridization buffer and MABT (100 mM maleic acid, 150 mM NaCl, 0.1% Tween-20, pH 7.5). Subsequently, baskets with embryos were transferred in MABT with 2% Roche blocking reagent and 20% heat-inactivated goat serum. Hybridized RNA was visualized with antidigoxigenin antibody coupled to alkaline phosphatase and BM purple substrate (both Roche, Mannheim, Germany). To initiate the color reaction, embryos were transferred to Petri dishes filled with the substrate and the reaction was allowed to proceed at room temperature in the dark for 2–5 days. *Xenopus leavis* embryos were obtained by hormone-induced egg laying and in vitro fertilization using standard methods [73]. Embryos were fixed in MEMFA and staged according to Nieuwkoop and Faber [46]. Whole-mount in situ hybridization was performed using digoxygenin-labeled antisense probes [73]. Xenopus shh construct was previously described (Ekker et al. [19, 20], and patched 2 construct was produced by PCR product from Xenopus cDNA corresponding to bp 2045–2751 (GenBank accession number: NM_001136166.1) using primer combination: XlPtch2L dir; ATTTCCACGTCACCCTCAGTCATT (forward) and GGTATCAGCCCCTTCTCATCCAC (reverse).

All embryos were photographed as whole mounts in Mowiol (Carl Roth, Karlsruhe, Germany) and embedded in Technovit 8100 s (Heraeus Kulzer, Wehrheim, Germany). Serial 5–10-mm sections were cut at sectional planes predefined in whole-mount views and photographed using bright-field illumination or Nomarski contrast.

## Results

### Axial expression of hedgehog genes in the chick

**Fig. 1:**
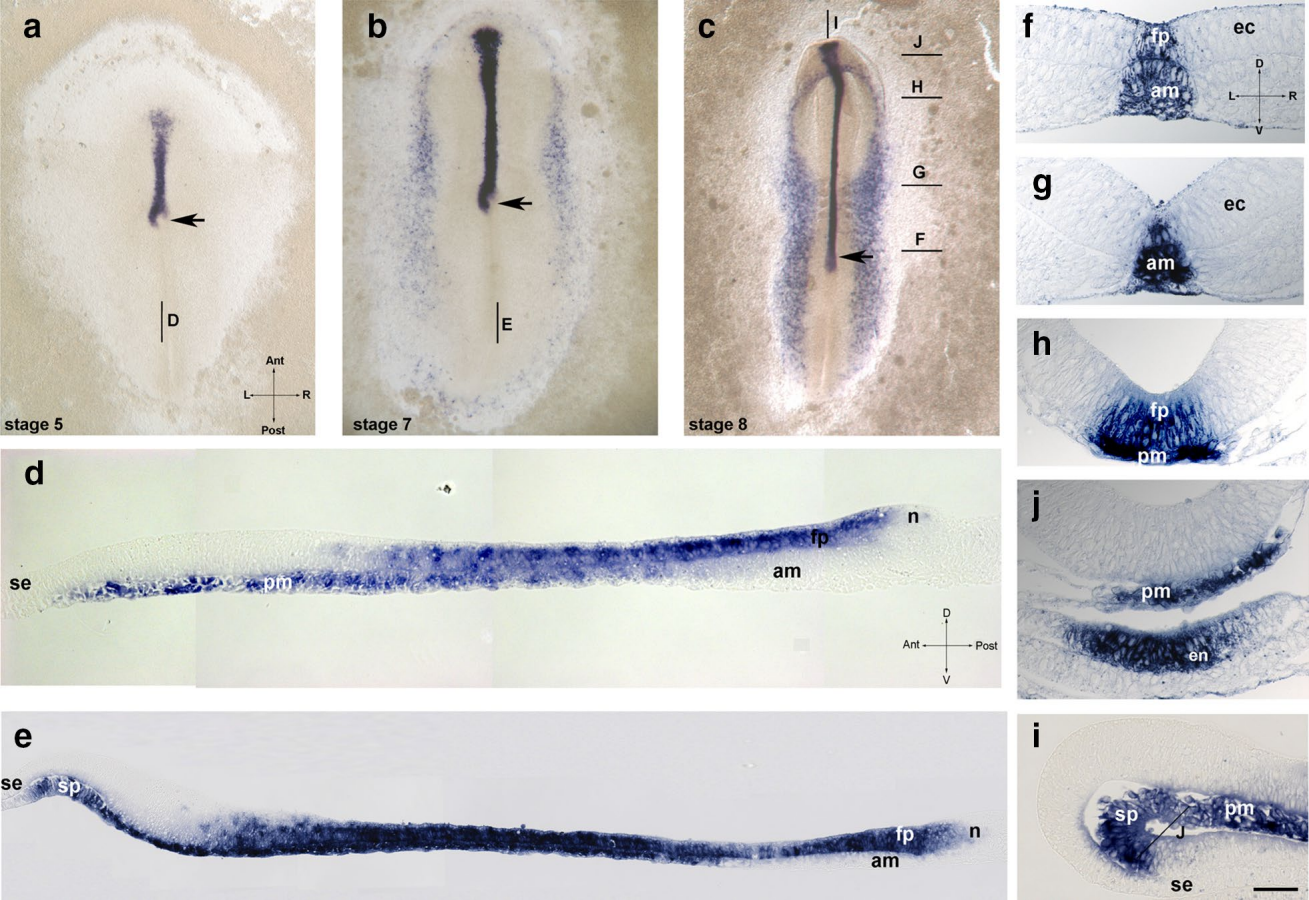
Temporal dynamics of *shh* expression in the chick. **a**–**c** whole-mount views of embryos at HH stage 5 (**a**), 7 (**b**) and 8 (**c**); median sagittal technovit sections of stage 5 (**d**) and stage 7 (**e**); **f**–**j** transversal sections of HH stage 8 embryos at the levels shown in **c**. **i** median sagittal section of stage 8 embryo. Labeling: *fp* floor plate, *am* axial mesoderm, *ec* ectoderm, *en* endoderm, *n* node, *pm* prechordal mesoderm, *sp* preoral gut (prospective area of Seessel’s pouch), *se* superficial ectoderm, arrow—position of the node. Intersecting arrows indicate anatomical axes: *A* anterior, *P* posterior, *L* left, *R* right, *D* dorsal, *V* ventral. Scale bar 400 µm (**a**–**c**), 75 μm (**e**) and 50 µm (**d**, **f**–**i**)

In HH stage 5 [29] chicken embryos, which are at the beginning of notochord formation and concomitant primitive streak regression, a *shh*-positive area appears as a stripe-like expression in the midline anterior to the node (Fig. 1a and Additional file 1: Fig. 1A), whereas the node domain displays progressive left–right asymmetry [86]. Importantly, analysis of technovit sections reveals that, at stages 5 (Fig. 1d and Additional file 1: Fig. 1D) and 6 (Fig. 1e), this *shh*-positive area lies posteriorly above a *shh*-negative or *shh-*weakly positive notochord. In the middle of the neural plate, both the midline mesoderm and the neuroectoderm are *shh* positive (Fig. 1D and Additional file 1: Fig. 1C), whereas in the area corresponding to the anterior neuroectoderm and the prechordal plate, the (upper) ectodermal layer is negative (Fig. 1D and Additional file 1: Fig. 1B). At HH stage 6 (Fig. 1b, e), expression extends in the posterior direction in both layers. Interestingly, at the level of the forebrain rudiment and head fold, the anterior expression is confined to epithelialized cells within the endoderm, whereas at stage 5 the expression in this area is confined to mesenchymal cells of prechordal mesoderm. Positive columnar cells within the endoderm display a columnar morphology and correspond to the preoral gut. In addition, at stage 6, scattered *shh*-positive mesenchymal cells are positioned at the anterior edge of the neural plate, thus forming a continuous border around the entire area pellucida (Fig. 1b). Analysis of *shh* expression at stage 8 (Fig. 1c, f–i) reveals expression along the whole notochord, in the prechordal mesoderm and in the anterior border of endoderm corresponding to so-called preoral gut and therefore to cells of the future Seessel’s pouch. The expression in the midline neuroectoderm remains strong and includes posterior regions, whereas expression in the forebrain area is not yet induced. In the node, *shh* domain is confined to the epiblast, while the adjacent notochord displays weak positivity. Summarizing *shh* is expressed in the prospective floor plate (except for its anterior part) in all serially sectioned chicken specimens (*N* = 37).

**Fig. 2:**
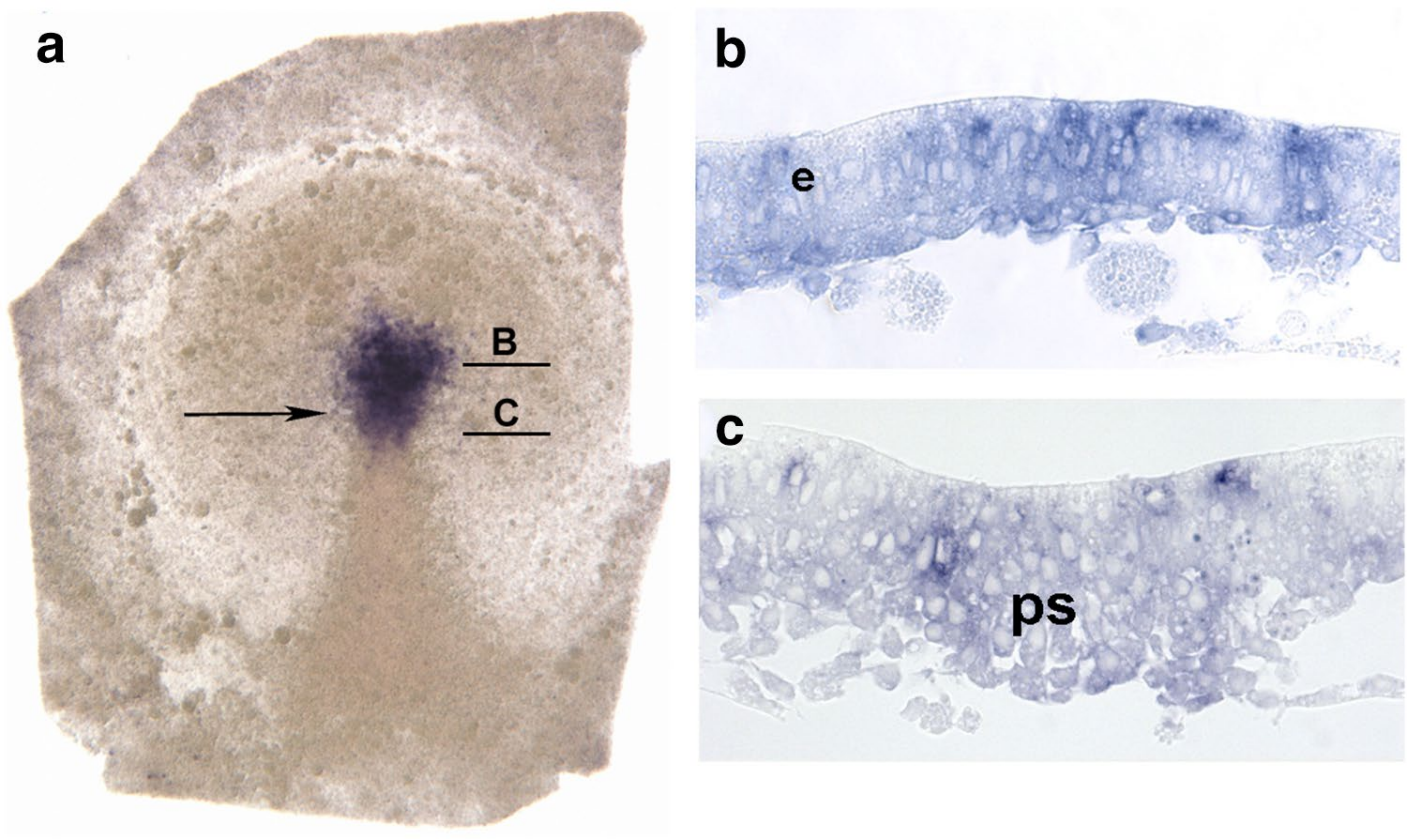
Early *shh* expression in the chick stage 2 +/3 − embryo. Arrow: position of the tip of the primitive streak

Since *shh* is expressed in the prospective floor plate from the beginning of the notochord formation as well as in the node, we asked whether its expression is initiated prior to node and prechordal mesoderm formation. Surprisingly, chick embryos are *shh* positive already between stages 2 and 3 (Fig. 2a). At this stage, the primitive streak appears as a density extending from the posterior pole to the center of the blastodisk and is in its posterior part wider and resembles an isosceles triangle [29]. The expression of *shh* is strong and related to the zone in the front of the medial thickening and in its anterior area. The area anterior to the primitive streak density has been shown to correspond to the prospective neuroectoderm, especially to the future floor plate, whereas the anterior portion of the primitive streak gives rise to the prechordal mesoderm and notochord [42, 79]. Transverse technovit sections near the posterior border of staining reveal a medial thickening with a groove (Fig. 2c), which corresponds to the anterior part of the primitive streak. Sections at the anterior level (Fig. 2b) reveal expression of heterogeneous intensity, which is confined to epiblast cells displaying a columnar epithelial shape. Additionally, single scattered mesenchyme-like positive cells representing early mesoderm are found under the epiblast. These cells correspond to local EMT areas, which were shown to occur at low intensity at this stage within the entire epiblast [94].

As *patched 2,* a marker of hedgehog signaling is confined to the midline neuroectoderm including forebrain after the beginning of the node regression [49, 51], we analyzed its expression prior to the node regression. *Patched 2* expression starts within the area pellucida in a uniform manner at stage 3 (not shown) and from stage 4 onwards is strongly expressed in the anterior epiblast (Additional file 2: Fig. 2). This expression corresponds to the ventral portion of the prospective neural plate and indicates active hedgehog signaling in the upper layer prior to the notochord formation.

**Fig. 3:**
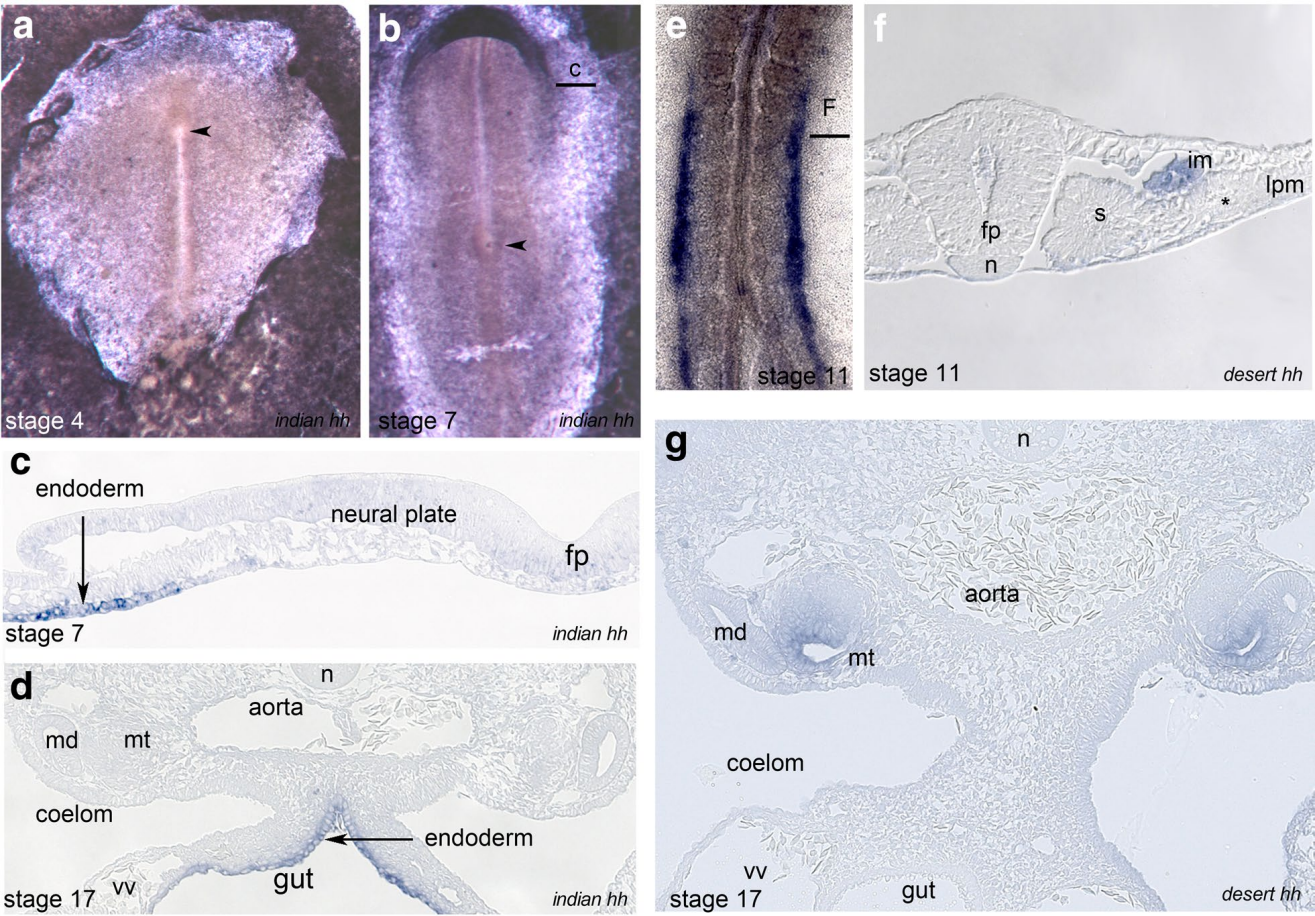
Expression of *Indian hedgehog* (*ihh*) and *desert hedgehog* (*dhh*) in the chick. **a**, **b** whole-mount views of *ihh* expression at HH stage 4 (**a**) and 7 (**b**). **c** transversal technovit section of embryo shown in **b**; **d** *ihh* expression in the gut endoderm (HH stage 17); whole-mount view (**e**) and section (**f**) of *dhh* expression in the dorsal part of intermediate mesoderm (HH stage 11); **f** *dhh* expression in the mesonephric tubules (HH stage 17). *N* notochord, *fp* floor plate, *im* intermediate mesoderm, *s* somite, *vv* vitelline vein, *md* mesonephric duct, *mt* mesonephric tubule, arrowhead—position of the node

Analysis of Indian hedgehog (ihh) and desert hedgehog (dhh) revealed no specific expression in the node, notochord or floor plate between HH stages 4 and 17. At stages HH4–HH5 *ihh* was weakly expressed in the anterior and lateral areas of zona pellucida (Fig. 3a) and at HH 6 and 7 lateral and anterior to the neural plate (Fig. 3b). Sections revealed strong expression in the endodermal layer which persisted at least until stage 17 (Fig. 3c, d). *Dhh* expression is first seen at stage 11 and is confined to two parallel narrow stripes lateral to paraxial somitic region (Fig. 3e). Sections confirmed expression in the dorsal domain of intermediate mesoderm (Fig. 3f). This expression is maintained and is also seen at stage 17 where *dhh* is expressed in the mesonephric tubules (Fig. 3g).

Summarizing, the presented data reveal that *shh* expression domain in the prospective chick neuroectoderm is initiated at early gastrula stage, displays continuity with the left-sided expression in the node epiblast and persists during early somitogenesis.

### Axial *shh* expression in the rabbit

**Fig. 4:**
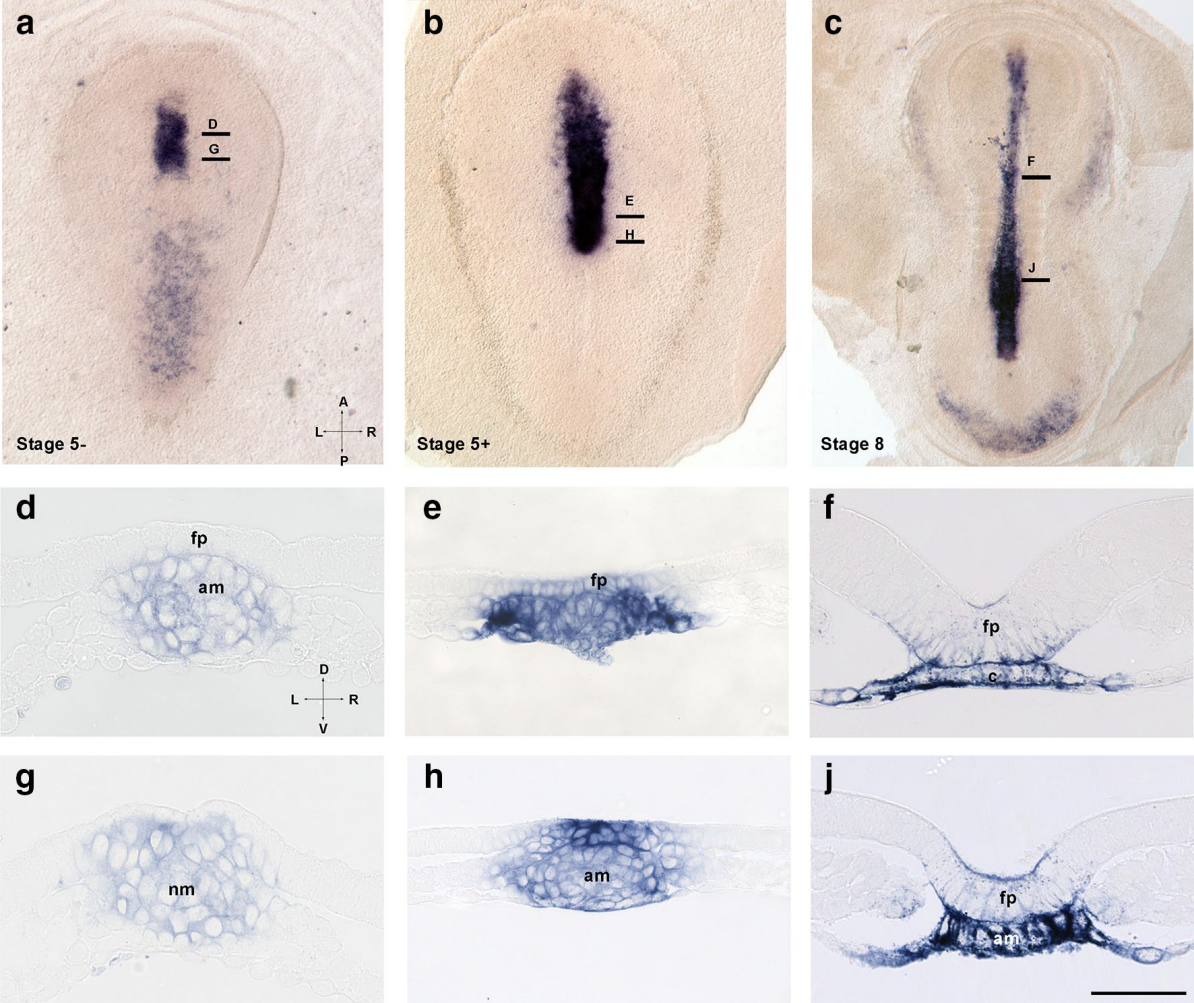
Temporal dynamics of *shh* expression in the rabbit embryo. **a**–**c** whole-mount views of stage 5 − (**a**), 5 + (**b**) and 8 (**c**) embryos. **d**–**j** transversal technovit sections of **a**–**c**. Levels of sections are shown in **a**–**c**. Labeling: *f* floor plate, *am* axial mesoderm, *nm* node mesoderm. Intersecting arrows in **a** indicate anatomical axes: *A* anterior, *P* posterior, *L* left, *R* right, *D* dorsal, *V* ventral. Scale bar 250 (**a**, **b**), 400 (**c**) and 50 µm (**d**–**j**)

Rabbit embryos display a symmetrical *shh* midline expression domain in the area of emerging notochord (Fig. 4a, b). Analysis of early stage 5 technovit sections anterior to the node shows strong expression in the emerging axial mesoderm (Fig. 4d) and no expression in the dorsal (neuroectodermal) layer, whereas within the node, *shh* is present in both layers (Fig. 4g). Similarly, at advanced stage 5 (Fig. 3e), the axial mesoderm reveals strong *shh* expression, while in the midline neuroectoderm, the expression is very weak with the exception of an area located immediately anterior to the node (Fig. 4h). Interestingly, the mesodermal expression is confined to the lateral area of the axial mesoderm, which has been shown to express *nodal* [69]. Remarkably, the notochord at the level anterior to the node may display an internal cavity indicating appearance of the notochordal canal in the rabbit in some specimens. Similar expression of *shh* is seen at stage 6 (not shown) and at stage 8 (Fig. 4c, f, j). At later stages, *shh*-positive cells are seen in the floor plate, notochord and in the dorsal endoderm. The data indicate that *shh* expression in the rabbit floor plate follows its expression in the notochord, similar to *shh* expression in the mouse [10].

### Axial *shh* expression in *Xenopus laevis*

**Fig. 5:**
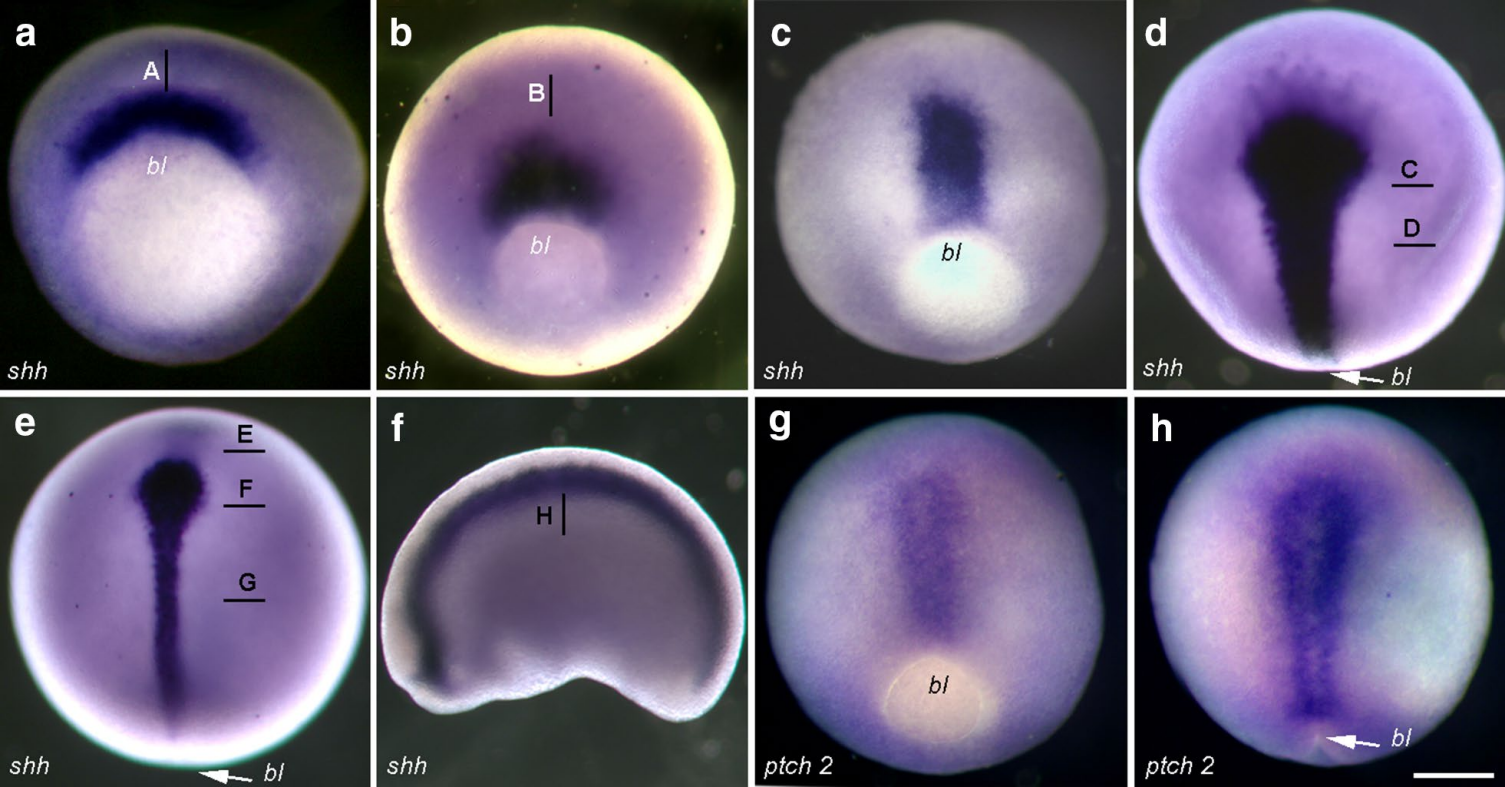
**a**–**f** temporal dynamics of *shh* expression in *Xenopus laevis* embryos between early gastrula and tadpole stages. **g**–**h** expression of *patched 2* at middle (**g**) and late (**h**) gastrula stage. *bl* Blastopore. Scale bar 200 µm

**Fig. 6:**
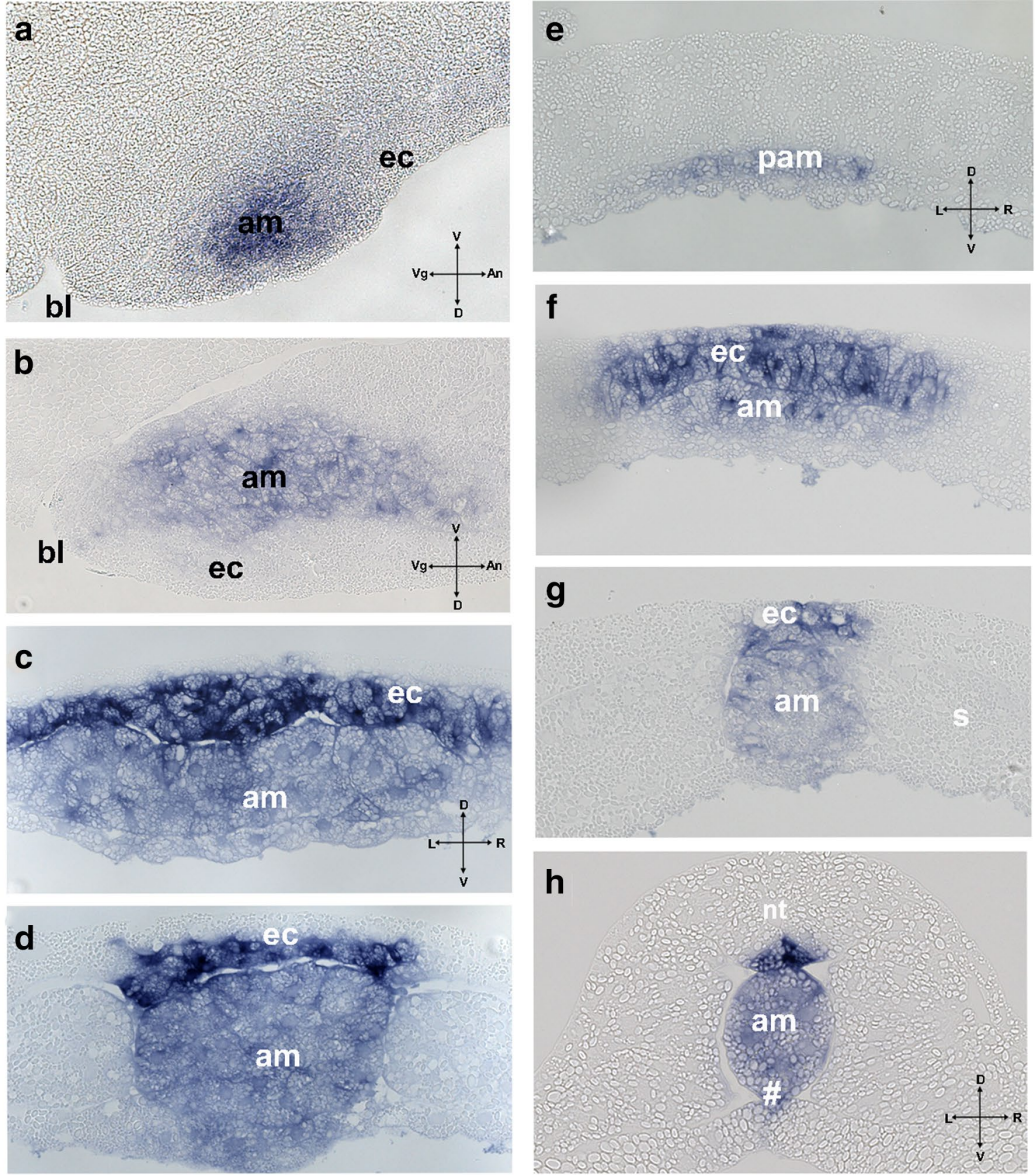
Technovit sections of *Xenopus laevis* embryos at early gastrula (**a**), mid-gastrula (**b**), late gastrula (**c**, **d**), early neurula (**e**–**g**) and tailbud stage (**h**) at levels shown in Fig. 3. Labeling: *ec* midline neuroectoderm, *am* axial mesoderm, *se* superficial ectoderm, *pam* preaxial mesoderm (leading edge), *s* somites, *nt* neural tube. Intersecting arrows in indicate anatomical axes: *A* animal, *Veg* vegetal, *L* left, *R* right, *D* dorsal, *V* ventral

In *X*. *laevis,* s*hh* expression starts at early gastrula stage with a sickle-shaped domain in the area above the dorsal blastopore (Fig. 5a). During gastrulation, this domain undergoes narrowing and elongation along the AP axis (Fig. 5b, c). Technovit sections reveal an early gastrula expression domain in the deep layer of the dorsal marginal zone, with expression absent from the dorsal lip and superficial mesoderm (Fig. 6a). The early *shh* domain corresponds to the area that is known to give rise to the axial mesoderm and is adjacent to the prospective neuroectoderm. At mid-gastrula, expression is confined to the deep involuted layer (Fig. 6b), and at the end of gastrulation, *shh* expression extends along the dorsal midline (Fig. 5d). Surprisingly, the sections reveal expression in the inner layer of the midline ectoderm and in the midline mesoderm (Fig. 6b, c). The width of the expression in both domains follows differences seen already in whole-mount views with a narrow posterior and wide anterior domain. During neurulation, *shh* expression undergoes further elongation and narrowing (Fig. 5e). Sections at stage 14 reveal expression in the inner neuroectoderm layer and weak expression in the rod-like notochord. In the anterior neuroectoderm, strong and wide expression is present in both inner cells, which display columnar morphology and in flat outer ectoderm cells. Midline mesoderm, which at this level forms a plate, also displays a strong scattered expression in epithelial-like cells. At the more anterior level (prospective superficial), expression of *shh* is present in the mesoderm, but excluded from the overlying ectoderm. At early tailbud stage (Figs. 5f, 6h), *shh* expression is confined to the floor plate, notochord, hypochord and at the level of the posterior notochord to the archenteron roof. In addition, we found that *patched 2* expression is specifically activated in the midline at mid-gastrula stages, suggesting activated hedgehog signaling already at this stage (Fig. 5g). Summarizing, neuroectodermal expression of *shh* is initiated at late gastrula/early neurula stages, whereas the mesodermal expression is initiated at early gastrula in the dorsal organizer area.

## Discussion

### *Shh* induction in the chick floor plate does not require a shh signal from the notochord

The first critical conclusion is deduced from the observation that after the beginning of the node regression (HH stage 5 -) *shh* is expressed in emerging prospective floor plate above *shh*-negative freshly laid down notochord. Moreover, a asymmetrical *shh* domain in the node remains continuous with the midline floor plate domain. At the same time, the notochord is continuous with a *shh*-negative right node shoulder [49]. This strongly suggests that during the investigated stages *shh* in the floor plate domain cannot be induced by shh protein secreted from the notochord. During further development, the elongating *shh* floor plate domain remains strongly positive at all studied stages, whereas the (ongoing emerging) notochord adjacent to the node remains *shh* negative until stage 8. These findings contradict the assumption that during the investigated stages, *shh*-expressing cells in the node lose *shh* expression when integrating into the floor plate and reacquire it after induction by shh from the notochord even though this assumption may apply for later stages. Delayed induction of *shh* in the posterior notochord may be promoted by HNF3 beta which is required for *shh* expression in mouse [4] and in contrast to shh is expressed in the freshly laid down notochord (see [66] and unpublished). Involvement of Indian and desert hedgehog in early inductive interaction is unlikely as they shown no midline expression.

Further conclusion concerns the “premature” initiation of *shh* expression at the late stage 2 in the epiblast in the front of the primitive streak as well as in prospective midline mesoderm in the anterior-most primitive streak. According to fate mapping data, the floor plate domain develops from epiblast adjacent and anterior to the node [22, 42, 62, 79]. Importantly, an area located at the early stage 3 at the anterior border of the prospective node corresponds to the *shh* domain that undergoes elongation during node regression and gives rise to floor plate cells within the neural tube with the exception of the forebrain, which emerges from more anterior region [42]. This indicates that the floor plate precursor cells are *shh* positive prior to mesoderm migration and suggests that floor plate induction may occur already at the beginning of gastrulation, similar to neural induction [81]. Strong expression of *patched 2* in the prospective neuroectoderm at stages 4 and 4 + further supports the “early” activated hedgehog signaling in the prospective floor plate.

Interestingly, fate mapping indicates a specific transformation of the floor plate domain in that the floor plate is recruited from a horseshoe-like domain anterior and lateral to the HH stage 4/4 + node (referred to as “full length primitive streak stage”, [23]). This distribution correlates remarkably well with the *shh* expression pattern: our previous analysis of *shh* expression between stages 4 − and 4 + revealed that the expression domain is first confined to the node, prenodal epiblast and pit mesoderm and thereafter to the scattered mesoderm cells anterior to the node, thus suggesting that at least a part of prechordal plate progenitor cells express *shh* continuously [86]. The mesodermal expression in the pit ceases at stage 4 + when the prechordal mesoderm has emerged from the node.

Our results require a modification of the so-called dual model for floor plate induction in the chick [50]. According to this model, the anterior floor plate including forebrain and midbrain does not require the notochord and is induced by rapid interaction of shh-negative precursor epiblast (also called area A) with migrating prechordal plate mesoderm, whereas prolonged influence of the notochord is required for floor plate induction in structures generated from HH stage 5 including the hindbrain and spinal cord [57]. Indeed, our data do not contradict the role of prechordal mesoderm and of hedgehog signaling in brain induction (see also [52]. However, as argued above, the proposed induction by the notochord [57] does not fit to continuous and persistent *shh* expression along the whole floor plate area which emerges after the beginning of node regression and during early somitogenesis (this study). The same time window has been suggested to limit the period of floor plate induction in both mouse and chicken [63].

However, considering the dynamic mode of floor plate generation [9] the involvement of shh from the notochord in the induction of the newly emerged floor plate at later stages cannot be excluded. In our view, the mode of floor plate induction in regions posterior to the cervical spinal cord should be investigated in detail to clarify whether there are even more numerous mechanisms of floor plate induction.

### Universal or divergent mechanisms of initial neural patterning

Further to the midline *shh* pattern in the chick embryo reported here and differing from data reported for mouse embryo [10, 18, 43], our analysis of *shh* expression in rabbit and *X*. *laevis* embryos revealed an additional divergence of spatiotemporal expression patterns among vertebrates (cf. Table 1 and Additional file 4: Table 1). Axial mesoderm in the rabbit has initial strong *shh* expression followed by expression in the floor plate similar to the mouse. Two findings, however, have not been reported previously: we found that strong *shh* expression is especially confined to the lateral domain that overlaps with the *nodal* domain, thus indicating possible involvement of hedgehog signaling in initiation of paramedian nodal domains [69]. Furthermore, our analysis revealed an initially short *shh* domain in the posterior floor plate. The latter can be explained by contribution of the *shh*-positive node to both notochord and posterior floor plate. Importantly, *patched* has been shown to be expressed in the rabbit prospective floor plate already at stage 5 indicating activated hedgehog signaling, while *HNF3*-*beta* expression was not detectable [24, 25]. Interestingly, Indian hedgehog does not display typical midline patterning in rabbit although it is expressed in the node and weakly in the short adjacent region of the notochord where it may be involved in hedgehog signaling, while desert hedgehog is not expressed at rabbit perigastrulation stages [8]. In summary, early *shh* expression in the rabbit midline axial mesoderm, together with signs of activated hedgehog signaling in the prospective floor plate above, supports the classical model, assuming that *shh* expression in the floor plate is induced by shh signaling from the notochord.

**Table 1:** Comparison of shh expression domains in equivalent axial structures of studied amniotes during notochord formation (HH/mammalian stage 5/6)

		Organizer level	Notochord level		
			Posterior	Mid	Anterior
Rabbit	Dorsal	+ (node-epibl)	− (floorpl)	− (floorpl)	− (floorpl)
	Ventral	+ (node-mes)	+ (not)	+ (not)	+ (not/pm)
Chick	Dorsal	+ (node-epibl)	+ (floorpl)	+ (floorpl)	− (floorpl)
	Ventral	− (node-mes)	− (not)	+ (not)	+ (not/pm)

Labeling: *not* notochord, *pm* prechordal mesoderm, *mes* mesoderm, *epibl* epiblast, *floorpl* floor plate

In *X*. *laevis*, ectodermal *shh* expression also follows the mesodermal expression pattern, and the prospective floor plate is *shh* positive already at the end of the gastrula above both emerging notochord and the wide spread *shh*-positive anterior axial (prechordal) mesoderm. Therefore, the induction of *shh* expression in the prospective floor plate of *X*. *laevis* is an early event and includes from its initiation the prospective brain region. Whether early *shh* expression in the floor plate is induced by dorsal mesoderm remains to be clarified although neither inhibition of hedgehog signaling with antisense morpholinos injected at the two-cell stage nor cyclopamine treatment from stage 8 was able to suppress substantially *shh* expression in the floor plate [54]. On the other hand, expression of *patched 2* at mid-gastrula indicates active hedgehog signaling (Fig. 5h). Interestingly, *shh* is expressed in the anterior midline leading edge mesoderm tissue known to be involved in head and heart formation, as well as in the analogous domain in the chick embryo (Fig. 1b). Other genes of the hedgehog family are not expressed in the midline of *X. laevis* embryos [19].

### The role of the notochord

Several experiments suggested that the notochord is a prerequisite for floor plate development [37, 59, 90, 91]. Node area excision in the gastrulating chick leads gives rise—apart from completely normally developed embryos [32, 61]—also to notochordless embryos, which display a.o. abnormal formation of neural folds, neural tube closure as well as the absence of typical floor plate cells [74]. At the same time, excision of the notochord piece in the chick anterior to the node at early stage 5 [50] does not affect *shh* expression in the midbrain and hindbrain. Although the excision of the node at 5–6 somite stage leads to midline abnormalities, regions with normal floor plate developed in notochordless areas [11]. Furthermore, the excision of the notochord at stage 9–10 leads to abnormal morphological development of floor plate in some areas of the neural tube, whereas other areas without the notochord do not display neural tube abnormalities; thus, it may be assumed that morphologically correct floor plate can develop without the underlying notochord [90]. An old study may help to interpret these observations: in the Triturus embryo with an absent notochord, the resulting abnormal floor plate was always associated with the fusion of somite material under the neural tube and, if somites were not fused, the neural tube developed a normal morphology [38]. A similar constellation may be true for the notochordless chick embryo [90] where the abnormal neural tube is seen above fused somites, whereas in other areas the neural tube resembles a normal shape.

### Organizer topography and the patterning of the neural tube

**Fig. 7:**
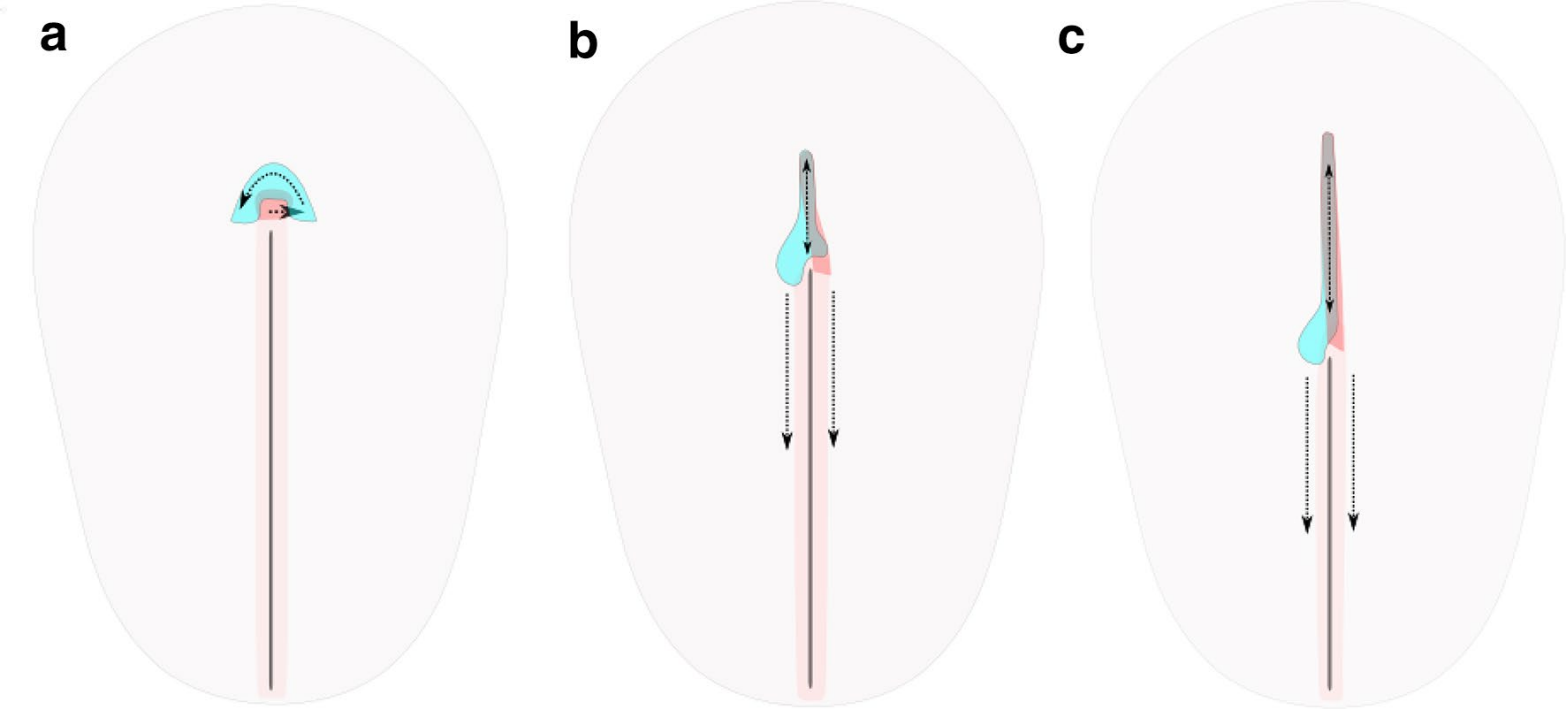
Axial morphogenesis during initial stages of primitive streak regression in the schematic dorsal view of chick embryo between stages 4 + (**a**), 5 − (**b**) and 5 + (**c**). Labeling: aqua blue—*shh* domain in the epiblast/ectoderm, red—notochord and its proposed progenitor domain, pink—primitive streak. Dotted arrows indicate directions of tissue displacement

On the basis of the results presented here, we hypothesize that divergent induction of *shh* expression in the midline neuroectoderm is due to divergent topography and morphogenetic movements during gastrulation. Evolution of vertebrate gastrulation has been proposed to be driven by increasing of yolk mass [5], which may lead via premature posterior activation of PCP pathway to stepwise transformation of circular blastopore into straight primitive streak of amniotes [7, 78, 80, 93]. This modified topography of organizer in turn may affect mechanisms of notochord formation. In the chick (Fig. 7), the notochord is laid down during so-called node (or primitive streak) regression [77], a process that is accompanied by a shift of the relative node position to the posterior pole of the embryo. Just prior to regression, the node tissue undergoes counterclockwise rotation [15, 27]. This rotation breaks the symmetry of the horseshoe-like *shh* domain located anterior to the node and transforms it into left-sided [15, 27, 39]. We propose that the notochord progenitor domain, which is located in the middle of the node [70] and at this stage does not express *shh* [86], is shifted to the right side (see Fig. 7). During primitive streak regression, tissues lateral to the node are shifted posteriorly, whereas the prospective neural plate undergoes elongation [68]. Therefore, the ectodermal *shh* domain also undergoes elongation which creates a stripe-like *shh* expression domain in the future floor plate (cf. Additional file 3: Fig. 3) which persists during studied stages and makes the inductive influence from the notochord unnecessary.

**Fig. 8:**
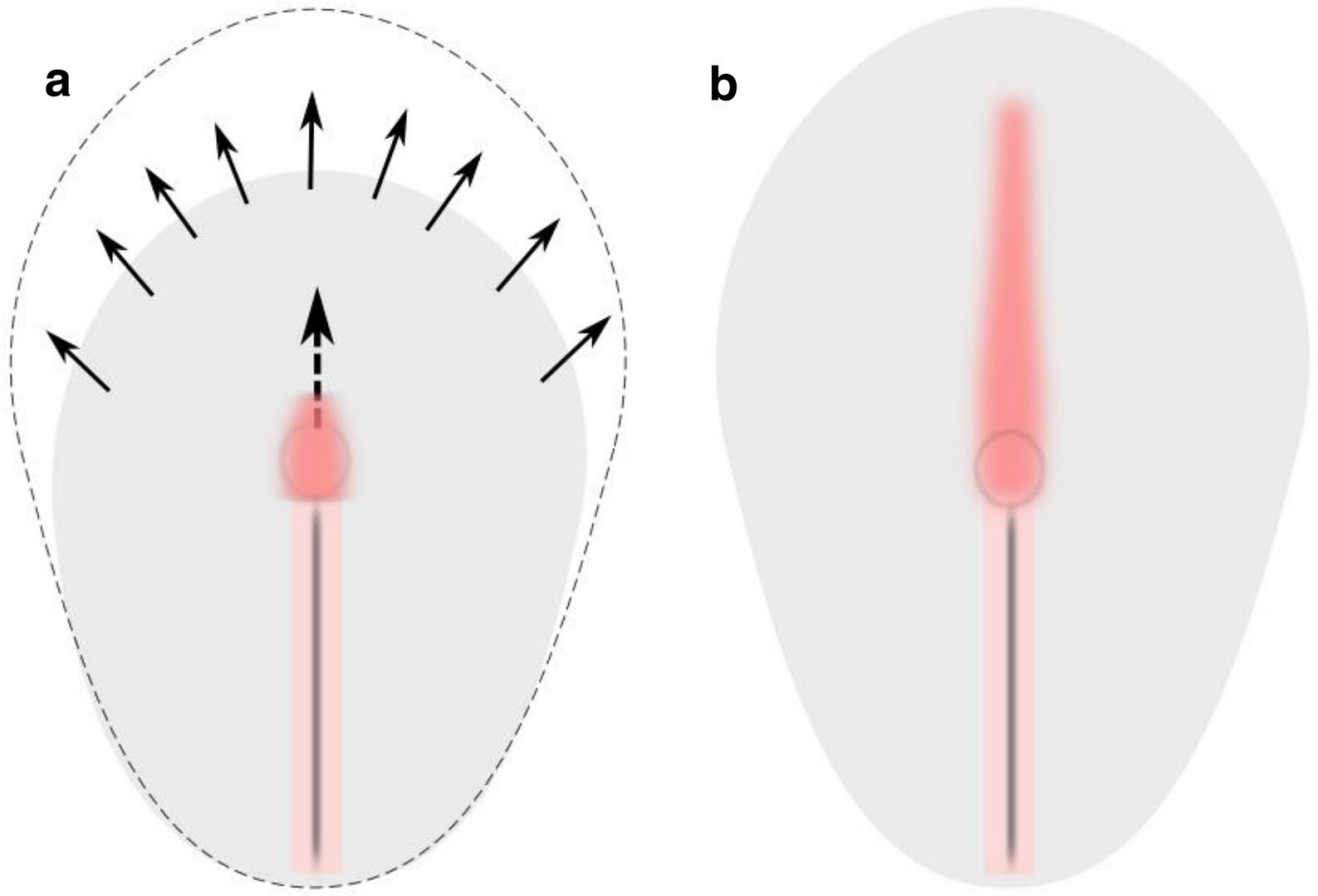
Expansion of prospective neural plate and growth of notochord from the node in the schematic dorsal view of rabbit embryo. **a** prior to the notochord formation (stage 4), **b** early notochord stage (stage 5). Labeling: arrows—growth of the blastoderm, dotted arrow—direction of notochord growth, red—notochord, pink—primitive streak, black—primitive groove

In the mouse and in the rabbit, however (Fig. 8), notochord formation is not accompanied by noticeable node regression [33, 96]. The major part of the murine notochord seems to be formed by convergent extension of the cells deriving from *shh*-positive node tissue. Whether the posterior part of the floor plate is also derived from the node is still a matter of debate. During notochord formation, the node remains symmetrical and bears symmetrical *shh* domain [25]. The axial mesoderm is *shh* positive along its whole length. The neural plate extension occurs by growth of tissue distant to the organizer [92]. Therefore, the induction of the floor plate requires external inductive stimuli, which are enabled by growth of the notochord beneath the prospective floor plate tissue (cf. Figure 8).

**Fig. 9:**
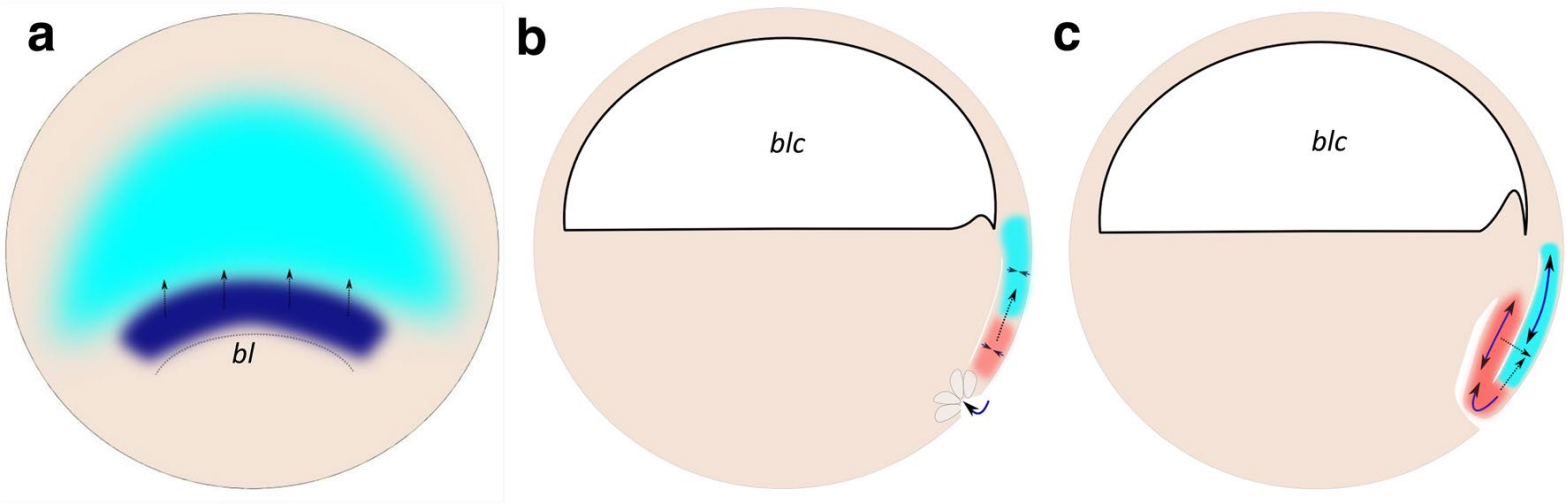
Schematic view of *Xenopus laevis* embryo during early and mid-gastrula: **a** dorsal view, **b** and **c** midline sagittal view. Labeling: red axial mesoderm, aqua blue—midline neuroectoderm, dark blue—*shh* expression domain, gray—bottle cells; blc—blastocoel, bl—blastopore, dotted arrows—direction of proposed induction, blue arrows—directions of morphogenetic movements, dotted curve in **a**—dorsal blastopore lip

In *X*. *laevis*, finally (Fig. 9), where the inductive interactions occur at an earlier stage, the notochord is generated by intercalation of dorsal mesoderm, which prior to invagination lies adjacent to the prospective neural tissue (Fig. 9). We are therefore tempted to speculate that the initial floor plate induction may occur at the stage where the neural tissue and mesoderm are arranged in close spatial planar neighborhood.

### Hedgehog expression and evolution of floor plate induction in chordates

In zebrafish, *shh* expression is initiated at 60% epiboly in the dorsal mesoderm, whereas already at 100% epiboly, *shh* is expressed in the midline anterior neuroectoderm above negative mesoderm, suggesting early induction [34]. Interestingly, neuroectodermal expression is initiated in the forebrain and progressively extends to the posterior pole, whereas in the chicken embryo expression extends in the opposite direction, hence indicating an additional divergence in the initiation of *shh* expression pattern. As already mentioned, the presence of the notochord is not required for zebrafish floor plate development, also suggesting that induction of *shh* in the zebrafish floor plate may be independent from the signal from the notochord and may occur at early stage. Importantly, other genes of hedgehog family are also expressed in the zebrafish midline structures. Moreover, this midline expression shows a segregated pattern: the expression of an orthologue of desert hedgehog is initiated at 50% epiboly in the embryonic shield and is at 90% epiboly expressed in the entire presumptive floor plate [21], whereas the expression of the Indian hedgehog orthologue is confined exclusively to the notochord [16]. Remarkably, expression of hedgehog family members at early stages of vertebrate development is highly divergent. In addition to axial expression of *shh,* a weak expression of Indian hedgehog was detected in the node and adjacent notochord of some mammals [8, 97], whereas in Xenopus [19] and chicken embryos (this report), the midline structures expressed sonic hedgehog only. These data are reminiscent of recurrent switching the function of paralogues shown for Snail1 and Snail2 in amniotes [41] and indicates functional divergence of hedgehog paralogues.

It has been suggested that the hedgehog family arose from single hedgehog gene also found in basal chordate amphioxus [72], whereas the *Ciona intestinalis* possesses two members (Ci-hh1 and Ci-hh2) that emerged in independent duplication events [84]. Analysis of *hedgehog* expression in Ciona shows expression of *Ci*-*hh2* in the ventral cells of the neural tube tissue prior to its expression in the notochord [84]. Initial *shh* expression in the ventral neural tube may be related to invariant development of cell lineage and early axis determination in ascidian embryogenesis, which functionally have lost organizer tissue although some inductive interaction and even regulative capacities have been observed [6, 47, 82, 83]. However, in amphioxus with its highly regulative development [87, 88] this dynamics may display an inverse spatiotemporal relation: although only whole-mount views from early stages were shown, the authors suggest that initially the *hedgehog *expression is localized in the presumptive endoderm and the notochord [72]. The situation in amphioxus may represent ancestral mode of floor plate induction.

**Fig. 10:**
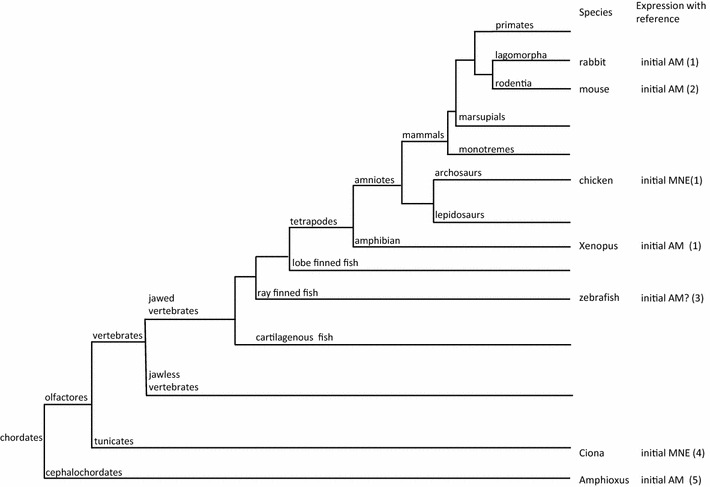
Initial *shh* expression in context of phylogeny of chordate. *MNE* midline neuroectoderm, *AM* axial mesoderm. References: 1—this report, 2—[10], 3—[34], 4—[84], 5—[72]

Comparison of reported data about initiation of axial hedgehog expression in chordates indicates evolutionary divergence in organizer tissues capable of floor plate induction and even patterning (Fig. 10). Phenotypic evolution has been proposed to be driven by developmental plasticity [56, 95] characterized by self-adjustment of developmental processes producing new phenotypic outcomes (cf. [55]. The role of such processes in the evolution of amniote gastrulation is supported by observation of experimentally induced modified gastrulation forms in rabbit and chicken embryo [1, 78, 93]. Altered gastrulation movements may facilitate changes in topography of axial organs and lead to the shift of expression domains, therefore driving the evolution of floor plate induction. In the next step, genetic accommodation and assimilation may be involved to ensure the stabilization of expression domains based on reciprocal molecular interactions [35, 45, 95].

Finally, developmental plasticity of floor plate inducing mechanisms is supported by recent findings of autonomous differentiation and floor plate marker induction in ES derived tissue: treatment of dorsalized neural tube organoids with retinoic acid leads to spontaneous symmetry breaking and dorsoventral patterning showing plasticity of dorsoventral patterning which may be induced by different triggers [30].

## Conclusions

Our comparative analysis of the spatiotemporal dynamics of *shh* expression indicates different sequences of *shh* induction in the prospective floor plate in several key vertebrate species and an evolutionary divergence of floor plate induction among vertebrates (Table 1 and Additional file 4: Table 1). Furthermore, it challenges (at least in the case of the chicken embryo) the model where the notochord is the primary source of hedgehog signaling in the floor plate (cf. Additional file 3: Fig. 3) by providing new observations and testable conclusions: *shh* expression in the chick prospective floor plate is initiated prior to mesoderm migration, there is no indication for downregulation of *shh* in the floor plate during early somitogenesis, and freshly laid down chick notochord is *shh* negative between HH stages 5 − and 8. Therefore, our data support and extend, by applying them to earlier stages, a similar proposal originally formulated by Le Douarine and co-workers [36, 85]. We further suggest that the mode of floor plate induction is related to the mechanism of notochord formation and propose that the initial induction of the floor plate marker *shh* in the future ventral cells of the neural tube evolves to adapt to the changing topography of interacting tissues which are the result of divergent morphogenetic movements in different embryo shapes prior and during gastrulation. Especially the “premature” expression of *shh* in the chick prospective floor plate seems to point to a spatiotemporal interdependence of axial organ formation, node regression, and left–right symmetry breaking, and to an involvement of early planar interaction within the epiblast. Further comparative and functional studies may now be designed to test this hypothesis.

## Additional files

**Additional file 1: Fig.** **1**. Expression of *shh* at the beginning of notochord formation. A: whole-mount view, B–D: transversal technovit sections at the levels shown in A.

**Additional file 2: Fig.** **2.** A. Expression of *shh* at stage 4 + , note absent reaction in the pit. B–D of *patched 2* expression at stages 4 − (B), 4 (C) and 4 + (D); note expression domain anterior to the node corresponding to the midline neuroectoderm. Arrow—the node area.

**Additional file 3: Fig.** **3.** Schematic transversal view of a chicken embryo at the level of the posterior notochord. A: ventral to dorsal floor plate induction in the classical view—*shh* is first expressed in the notochord and induces *shh* in floor plate. Shh protein from both sources forms a gradient. B: modified model demonstrating early shh expression and shh gradient formation in the floor plate. Labeling: pink—notochord, blue—floor plate, green—hypoblast/endoderm, brown—paraxial mesoderm, arrows—gradient formation and induction.

**Additional file 4: Table** **1.** Summary of *shh* expression in equivalent axial structures in chicken, rabbit and *Xenopus laevis*.
