# Supplementary figures and images for: Divergent axial morphogenesis and early shh expression in vertebrate prospective floor plate
Source: EvoDevo. 2018 Jan 31;9:4. doi: 10.1186/s13227-017-0090-x (PMC5791209; doi:10.1186/s13227-017-0090-x)

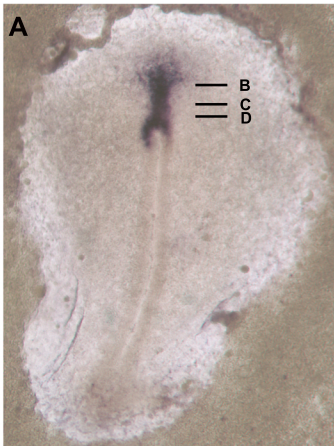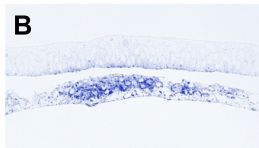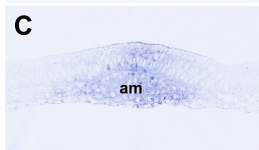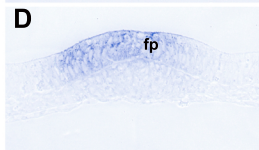

Supplement: Supplementary file 1 — Additional file 1: Fig. 1. Expression of shh at the beginning of notochord formation. A: whole-mount view, B–D: transversal technovit sections at the levels shown in A. [file 13227_2017_90_MOESM1_ESM.pdf]

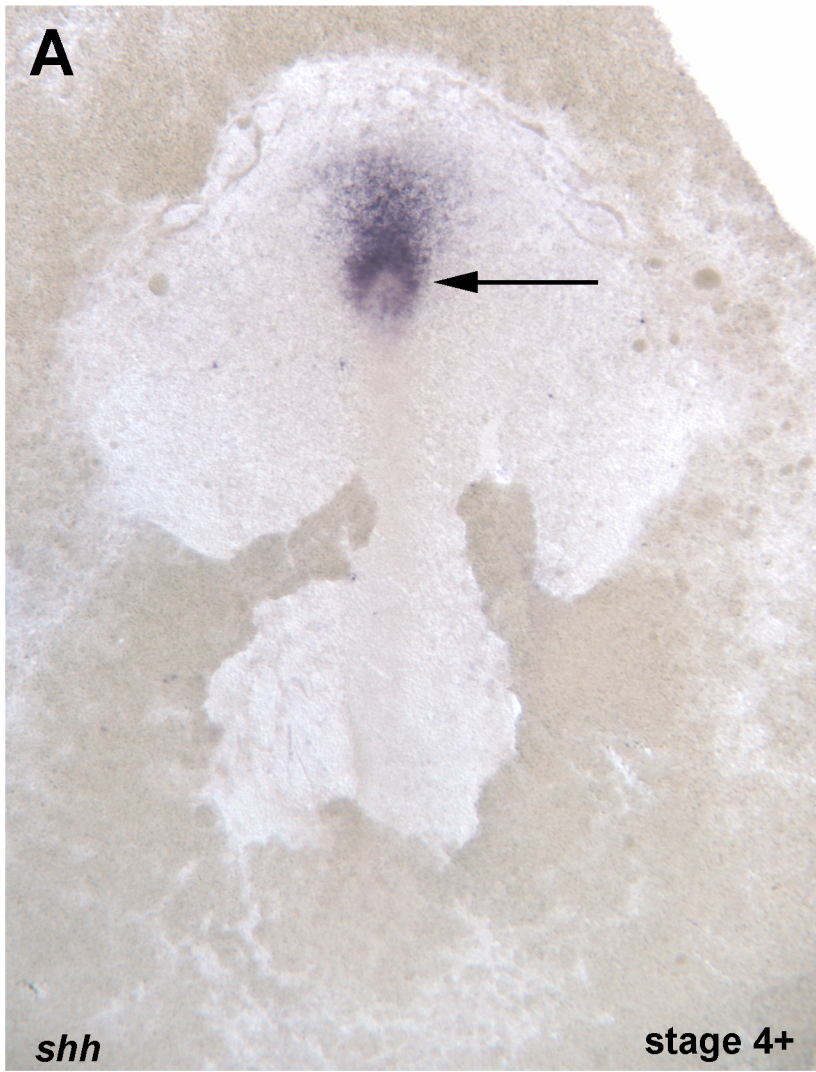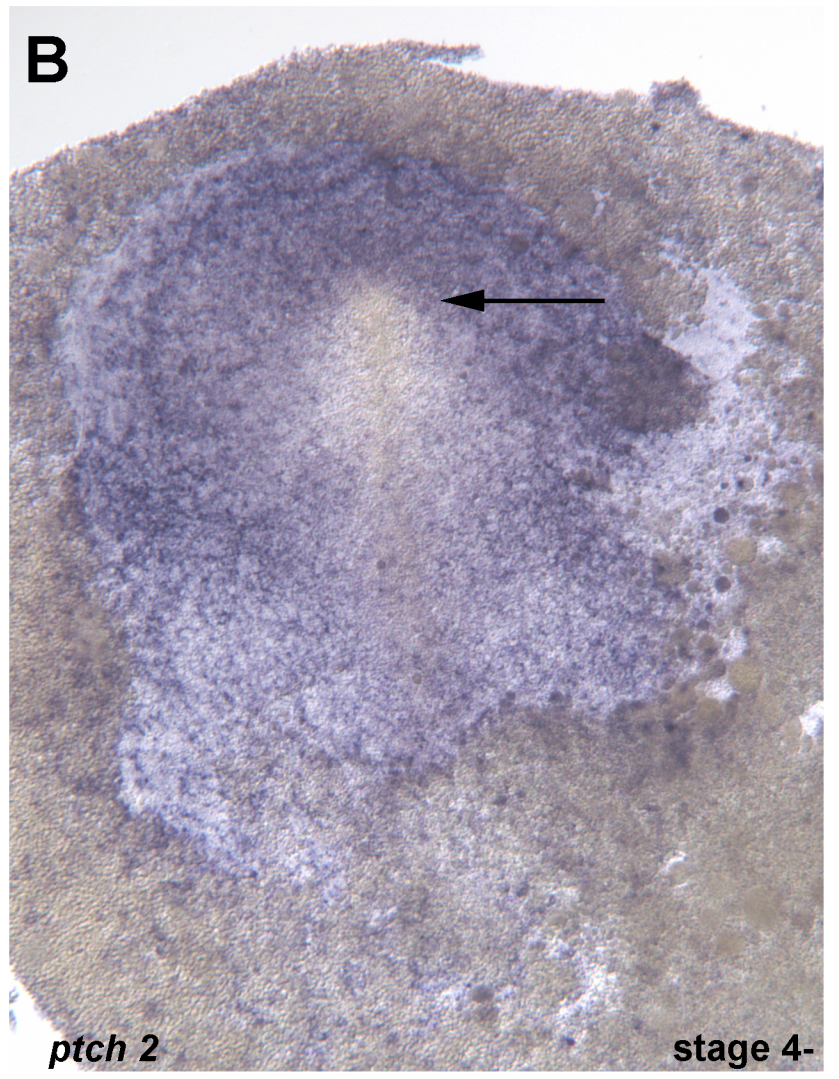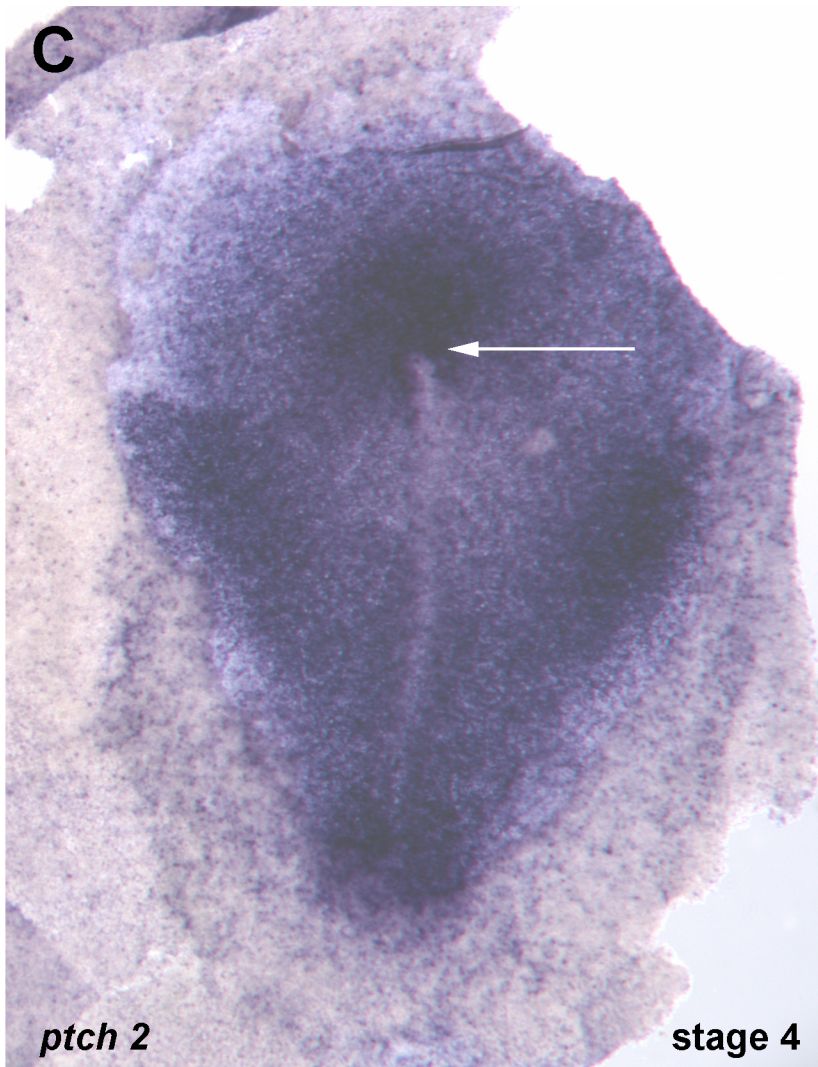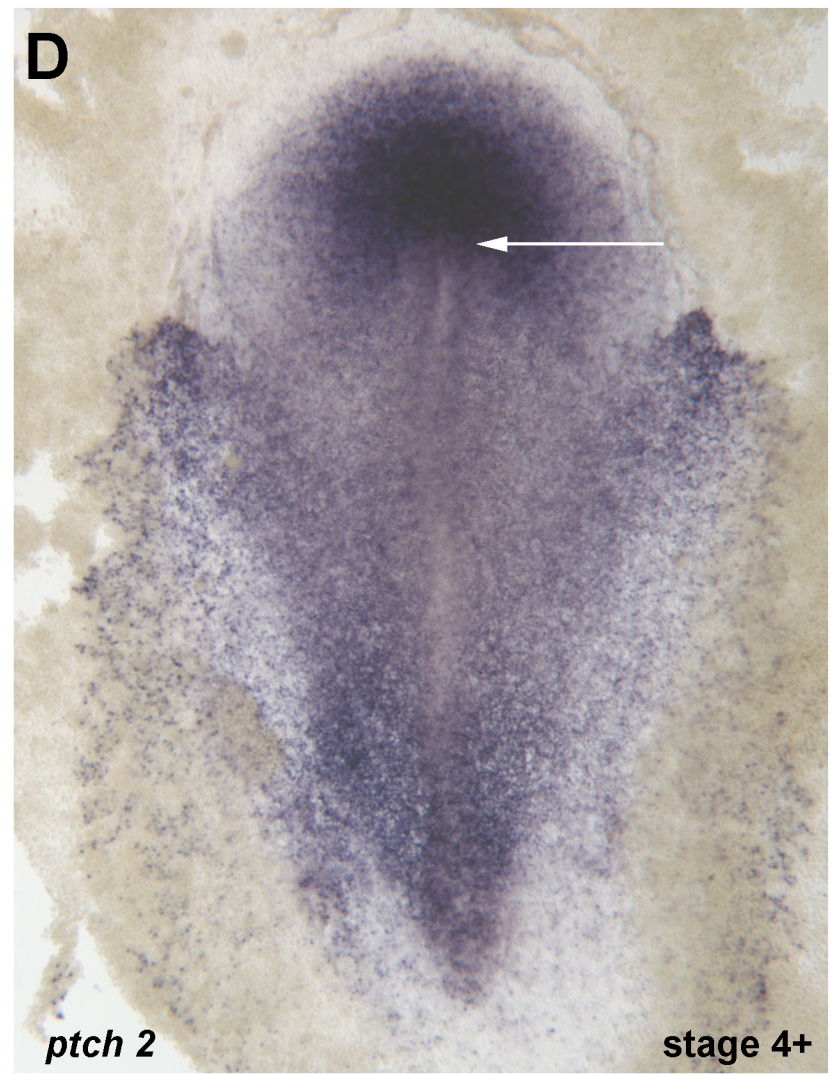

Supplement: Supplementary file 2 — Additional file 2: Fig. 2. A. Expression of shh at stage 4 + , note absent reaction in the pit. B–D of patched 2 expression at stages 4 − (B), 4 (C) and 4 + (D); note expression domain anterior to the node corresponding to the midline neuroectoderm. Arrow—the node area. [file 13227_2017_90_MOESM2_ESM.pdf]

**A**

dorsal

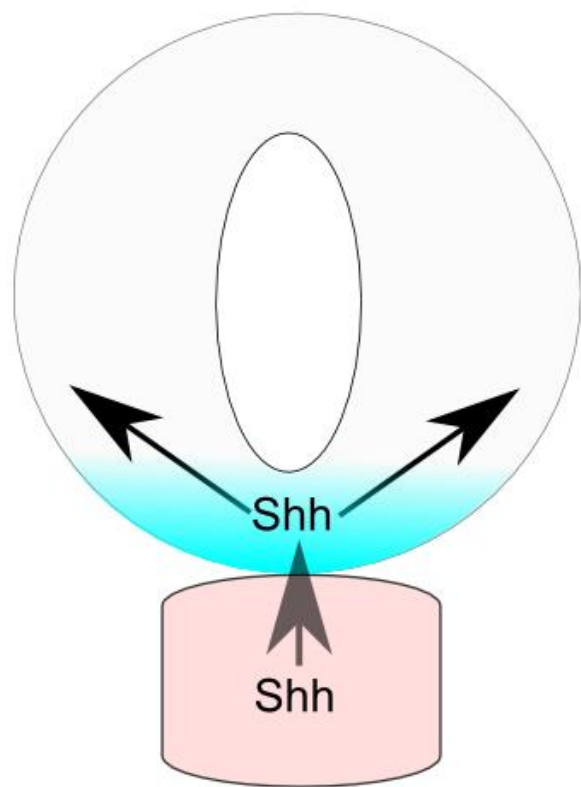

ventral

**B**

left

right

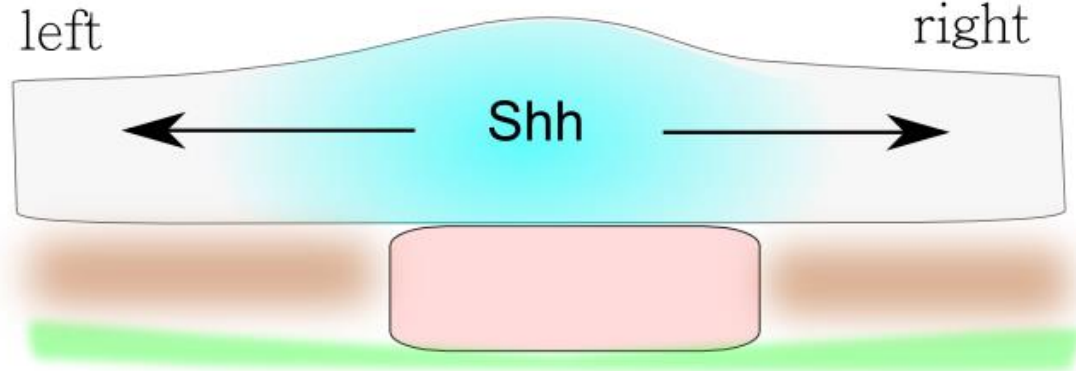

Supplement: Supplementary file 3 — Additional file 3: Fig. 3. Schematic transversal view of a chicken embryo at the level of the posterior notochord. A: ventral to dorsal floor plate induction in the classical view—shh is first expressed in the notochord and induces shh in floor plate. Shh protein from both sources forms a gradient. B: modified model demonstrating early shh expression and shh gradient formation in the floor plate. Labeling: pink—notochord, blue—floor plate, green—hypoblast/endoderm, brown—paraxial mesoderm, arrows—gradient formation and induction. [file 13227_2017_90_MOESM3_ESM.pdf]
