# Supplementary material for: Divergent axial morphogenesis and early shh expression in vertebrate prospective floor plate
Source: EvoDevo. 2018 Jan 31;9:4. doi: 10.1186/s13227-017-0090-x (PMC5791209; doi:10.1186/s13227-017-0090-x)
Supplement: Supplementary file 4 — Additional file 4: Table 1. Summary of shh expression in equivalent axial structures in chicken, rabbit and Xenopus laevis. [file 13227_2017_90_MOESM4_ESM.pdf]

Shh expression domains in equivalent structures

species	domain	expression
chicken	dorsal organizer: the pit area of the node	positive prior to mesoderm formation; negative after stage 4+
chicken	dorsal organizer: anterior and lateral node	initially positive; right side: negative after stage 5-
chicken	notochord	posterior: negative at the beginning of notochord formation; anterior: positive
chicken	floor plate/midline neuroectoderm	positive in the middle and posterior area; negative in the forebrain
Xenopus	dorsal blastopore /organizer	positive at early gastrula
Xenopus	notochord	positive at studied stages
Xenopus	floor plate/midline neuroectoderm	negative at gastrula stages; positive from neurula incl. forebrain
rabbit	node	positive at studied stages
rabbit	notochord	positive at studied stages
rabbit	floor plate/midline neuroectoderm	negative at the begin of the notochord formation, later positive
